# Sustainable Polyurethane Networks Based on Rosin with Reprocessing Performance

**DOI:** 10.3390/polym13203538

**Published:** 2021-10-14

**Authors:** Jiawei Li, Weiming Yang, Zhao Ning, Bin Yang, Yanning Zeng

**Affiliations:** Key Laboratory of New Processing Technology for Nonferrous Metal and Materials, Ministry of Education, College of Material Science and Engineering, Guilin University of Technology, Guilin 541004, China; zjljw1998@163.com (J.L.); ywm18378330339@163.com (W.Y.); ningzhao19971016@sina.com (Z.N.); a18832111836@163.com (B.Y.)

**Keywords:** rosin, polyurethane vitrimer, reprocessing, self-healing

## Abstract

Rosin is an abundant natural product. In this paper, for the first time, a rosin derivative is employed as a monomer for the preparation of polyurethane vitrimers with improved properties. A novel rosin-based polyurethane vitrimers network was constructed by the reaction between isocyanates (HDI) as curing agent and monomers with alcohol groups modified from rosin. The dynamic rosin-based polyurethane vitrimers were characterized by FTIR and dynamic mechanical analysis. The obtained rosin-based polyurethane vitrimers possessed superior mechanical properties. Due to the dynamic urethane linkages, the network topologies of rosin-based polyurethane vitrimers could be altered, contributing self-healing and reprocessing abilities. Besides, we investigated the effects of healing time and temperature on the self-healing performance. Moreover, through a hot press, pulverized samples of 70%VPU_OH_ could be reshaped several times, and the mechanical properties of the recycled samples were restored, with tensile strength being even higher than the of that of the original samples.

## 1. Introduction

Polyurethane (PU) thermosets, which are the sixth largest class of synthetic polymers, have found wide applications in the fields of elastomers, foams, adhesives, coatings, and structural components [1,2]. PU thermosets are typically prepared by the reaction of isocyanates and monomers with alcohol/amino groups, leading to the formation of urethane groups. The various mechanical properties of PU thermosets derive from the diverse structural availability of both alcohol and isocyanate monomers. The desirable mechanical properties of PU thermosets come from the combination of “hard” and “soft” domains. The “hard” domains formed by urethane groups which are capable of hydrogen bonding provide stiffness and toughness, and the “soft” domains from aliphatic monomers supply flexibility to the material. However, it is exceptionally difficult to recycle the covalently cross-linked networks of PU thermosets. Though mechanically grinding approaches have been employed to downcycle PU thermosets into fillers to enhance adhesive composites [3], chemical recycling of PU thermosets remains a challenge.

Several strategies to obtain malleable PU thermosets have been reported. One of strategy involves the introduction of dynamic Diels–Alder (DA) adducts into PU networks by grafting functional groups onto the main chains [4,5,6]. Another strategy involves PU cross-linking with dynamic covalent bonds, such as urea bonds [7,8], reversible C−C bonds [9], disulfide bonds [10,11], aromatic Schiff base bonds [12], and imine bonds [13]. The major limitations of the two approaches relate to the cost of the materials themselves and the changes in the properties of the PU thermosets. Therefore, controlling the dynamic nature of urethane linkages is a more straightforward way to recycle PU thermosets, because the essential carbamate bonds are exchangeable. The innovative work by the Dichtel group [14] has explored PU thermosets with covalently adaptable networks (CANs) providing a reprocessing capability which is attributed to the dissociation of urethane linkages to isocyanates and alcohols. However, PU CANs cannot maintain the integrity of the network, which leads to inconstant urethane linkages. PU vitrimers with exhibit a distinct dimensional stability and network integrity, because the crosslinked bond does not break until a new bond is formed, which makes the network permanent and dynamic. PU vitrimers show a gradual viscosity decrease upon heating, which is a distinctive character of vitreous silica. Furthermore, they are repairable, malleable, weldable and reprocessable by compression molding, due to thermally induced dynamic interexchange reactions. Therefore, PU vitrimers with dynamic urethane linkages have been developed to obtain new properties of polymers, including shape memory [15], self-healing [16,17], malleability [18,19], and reprocessability [20]. In brief, transcarbamoylation reaction in various PU thermosets have been researched to facilitate reprocessability, because the exchange reaction in the unique urethane linkages can occur with two different mechanisms. For conventional polyurethanes, the dissociative mechanism is the predominant one, while it is found that to obtain PU vitrimers, the effective approach is to form polyhydroxyurethane by introducing many hydroxyl groups in polyurethane [15,21]. Besides, another key factor regarding PU vitrimers system is the presence of an appropriate catalyst which contributes to transcarbamoylation reactions, providing reprocessing ability [22,23]. As a conclusion, it is crucial to obtain polyhydroxyurethane and add an appropriate catalyst for the synthesis of PU vitrimers.

On the other hand, most of the PU vitrimers are obtained from nonrenewable petroleum resources. With the rising awareness of the need of environmental protection, seeking alternative biomass resources for the synthesis of PU vitrimers has become significant for sustainable green materials. Lei group synthesized multifunctional PU vitrimers from renewable castor oil which can be reprocessed at 180 °C in 2 h in the presence of dibutyltin dilaurate (DBTDL) catalyst [24]. Qiu group reported the usage of lignin in cross-linked PU networks to maintain excellent mechanical performance after hot reprocessing [25]. Among various types of biomass resources, rosin is a good candidate for PU vitrimers preparation because its hydrophenanthrene ring structure may enhance polyurethane properties, especially its hardness [26]. The hydrogen phenanthrene ring structures of rosin acids are advantageous to obtain the desired thermal and mechanical properties of the derived thermosets. For example, improvements in *T_g_* of cured epoxidized soybean oil are significantly influenced by rosin-based derivatives used as the curing agent [27,28], and cured epoxies employing rosin as the curing agent display a higher *T_g_* than the commercial monocyclic analogs [29,30]. In this study, high mechanical properties and reprocessable PU vitrimers based on rosin are fabricated for industrial sustainable development.

## 2. Experimental

### 2.1. Materials

Rosin (acid number 166 mg KOH g^−1^, softening point 76 °C) was kindly supplied by Guilin Xingsong Forest Chemical Co., Ltd. (Guilin, China). Maleic anhydride (99%), DBTDL (95%), and HDI were purchased from Aladdin (Shanghai, China). Toluenesulfonic acid was purchased from Macklin (Shanghai, China). Pentaerythritol was purchased from Sinopharm Chemical Reagent Co., Ltd. (Shanghai, China). Tetrahydrofuran (THF), acetic acid, and dimethylbenzene were purchased from XiLong scientific Co., Ltd. (Shantou, China).

### 2.2. Synthesis of Maleopimaric Anhydride (MPA)

**Scheme 1:**
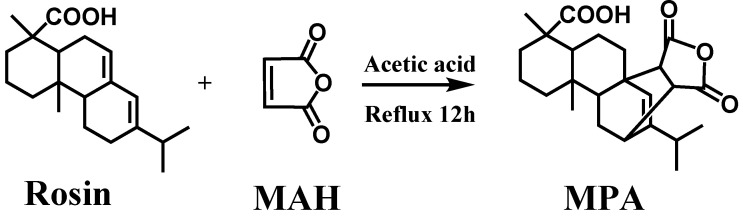
Schematic diagram of the synthesis steps of MPA.

MPA was synthesized as reported [31] in Scheme 1. Rosin (50.0 g, 0.16 mol) was introduced into a 250 mL three-necked round bottom flask equipped with a mechanical stirrer, a thermometer, and a reflux condenser, then was heated to 180 °C and let react for 3 h under a N_2_ atmosphere to complete the isomerization of the abietic structure to a levopimaric structure. The reaction mixture was cooled to 120 °C; then acetic acid as the solvent (150 mL), maleic anhydride (24.0 g, 0.24 mol), and toluenesulfonic acid as a catalyst (2.5 g) were added, and the reaction mixture was refluxed for 12 h. A yellow solid crude product was obtained, which was further recrystallized with acetic acid to give white crystals of pure MPA (yield: 62%).

### 2.3. Synthesis of an Esterified Adduct of MPA with Pentaerythritol (PEMPA)

**Scheme 2:**
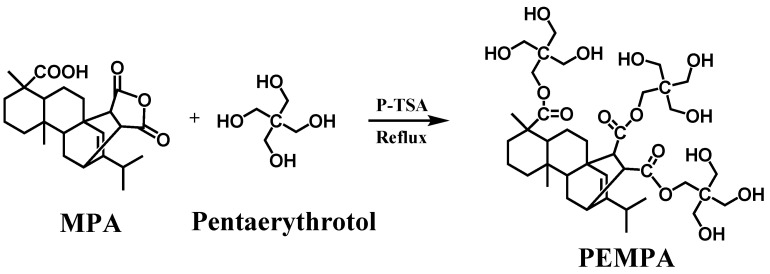
Schematic diagram of the synthesis steps of PEMPA.

PEMPA was prepared as described [32] in Scheme 2. In a 250 mL flask equipped with a mechanical stirrer, a Dean–Stark device, and a thermometer, we mixed MPA (20 g, 0.05 mol), pentaerythritol (20 g, 0.15 mol), toluenesulfonic acid as a catalyst (accounting for 1% of the total mass of the reactants), and xylene as the solvent (40 mL). The reaction mixture was refluxed until 0.1 mole of water was collected. A light-yellow solid was obtained and washed with hot water to remove unreacted pentaerythritol (yield: 64%).

### 2.4. Synthesis of the VPU_OH_ Cross-Linking Network

**Figure 1:**
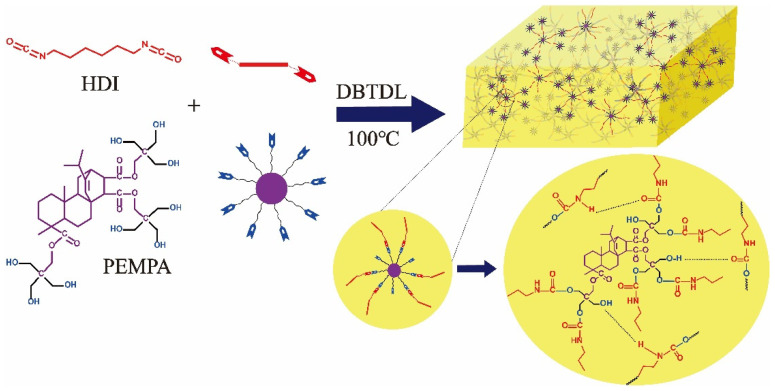
Synthesis of the VPU_OH_ cross-linking network.

A typical reaction of PEMPA and HDI to fabricate VPU_OH_ networks is shown in Figure 1. HDI and a catalytic amount of DBTDL were added into the THF solution of PEMPA under accelerating stirring until they were dissolved. The mixture was quickly transferred to a release paper mold and let stand at r. t. for 12 h. Then it was sequentially cured at 60, 80, and 100 °C for 4 h. The HDI content was 30, 40, 50, 60, 70, and 80 mol% relative to PEMPA; the abbreviation of x%VPU_OH_ refers to the cured PEMPA with x mol% of HDI.

### 2.5. Characterizations

Fourier-transform infrared (FTIR) spectra were collected using a Nicolet 205 FTIR spectrometer (Waltham, MA, USA) from 500 to 4000 cm^−1^ by the KBr tablet method. Stress relaxation tests were carried out using a TA Q800 instrument for dynamic mechanical analysis (DMA). Stress relaxation experiments were conducted by monitoring the stress decay at a constant strain of 5% after equilibrating at the required temperatures for 20 min. The loss angle and storage modulus of the material were measured by a TA Q800 (New Castle, DE, USA); the VPU_OH_ samples (20.0 mm × 5.0 mm × 1.0 mm) were tested from 25 to 200 °C (heating rate = 3 °C min^−1^) with a frequency of 1 Hz. The glass transition temperature (*T_g_*) was obtained from the inflection point of the curve, and the crosslink density (*V_e_*) was obtained from the storage modulus curve. The thermal decomposition behavior of the PEMPA and VPU_OH_ series was examined by thermogravimetry (TGA) at a heating rate of 10 K/min in a nitrogen atmosphere from 35 to 800 °C with a TG 209 (NETZSCH, Selb, Germany). NT-MDT atomic force microscopy was used to observe the phase structure of 70%VPU_OH_ samples in the tapping mode with a force constant of 5.5 N/m and a resonance frequency of 219 kHz. The mechanical performance test employed the UTM4503SLXY universal tensile testing machine of Shenzhen Sansi aspect Technology Co., Ltd. (Shenzhen, China), with 5 mm/min tensile rate; the rectangular samples (50 mm × 8 mm × 1 mm) were aged for three days in a desiccator at ambient temperature prior to testing. Testing was repeated three times testing for each sample. The error bar is the standard deviation obtained from the square root of the arithmetic mean of the squares of the deviations. The original sample used for the mechanical property test was 40 mm, and the length of the fracture sample was 64.2–87.8 mm.

Self-healing experiments were recorded using a polarizing optical microscopy (POM) equipped with a heating stage and a UCMOS05100KPA (P/N: TP605100A) microscope camera (ToupTek Photonics Co., Ltd., Hangzhou, China). The strip samples (70%VPU_OH_) were cut using a razor to obtain cracks. The cracked samples were heated in an oven at a controlled temperature (140, 150, or 160 °C) for 2–4 h, and thereafter the cracks were observed using POM.

Welding was performed with two rectangular samples of 70%VPU_OH_ (25.0 mm × 5.0 mm × 1.0 mm). They were held together with a superimposed length of 8 mm for s 3 h at 160 °C. A good contact was ensured after heating. To evaluate the welding strength, the tests was conducted by pulling down weights (3 kg).

To study shape memory, the sample strip (70%VPU_OH_) was brought to 120 °C, bent into different shapes using an external force, and finally cooled down to room temperature. Digital photos of the strip sample before and after reshaping were recorded.

The reprocessing flake sample (70%VPU_OH_) was grinded into powder using a file. A hydraulic plate vulcanizer (ZS-406B-30-300, Dongguan Zhuosheng Machinery Equipment Co., Ltd., Dongguan, China) was used as the reprocessing equipment, and the obtained sample powder was added into the reprocessing mold under 5 MPa pressure. Reprocessing was performed at 160 °C for 30, 60, or 90 min; the samples were subjected to three cycles of reprocessing.

## 3. Results and Discussion

### 3.1. Covalent Cross-Linking of VPU_OH_ Using HDI

**Figure 2:**
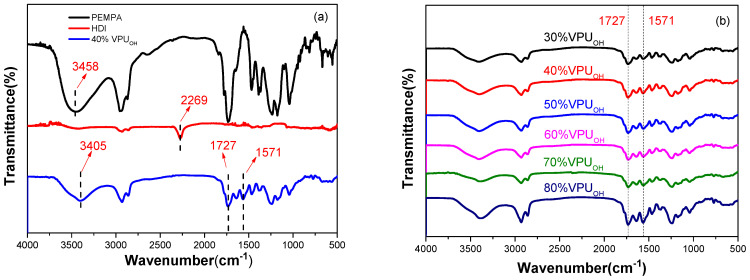
FTIR spectra: (**a**) HDI, PEMPA, and 40%VPU_OH_, (**b**) VPU_OH_ with different HDI content.

The VPU_OH_ networks were fabricated based on the chemical reaction between PEMPA with −OH groups and HDI with −N=C=O functional groups. They reaction was confirmed by FTIR measurements of HDI, PEMPA, and 40%VPU_OH_. The related spectra are shown in Figure 2a. In the spectrum of PEMPA, absorption at 3458 and 1727 cm^−1^ was ascribed to the stretching vibrations of −OH and C=O, respectively [33]. In the case of HDI, the absorption peak at 2269 cm^−1^ originated from the stretching vibrations of N=C=O [34]. The absorption around 1500–1600 cm^−1^ was attributed to the distortion vibrations of N–H and the stretching vibrations of C–N of the urethane bond, and the peak at 3428 cm^−1^ was assigned to the stretching vibrations of N–H, in the spectrum of 40%VPU_OH_ [35]. In comparison with the spectrum of HDI, the peak at 2269 cm^−1^ disappeared in the spectrum of 40%VPU_OH_, because abundant −OH groups in PEMPA reacted with N=C=O groups in HDI, so that N=C=O was not present in 40%VPU_OH_. Meanwhile, compared with the spectra of PEMPA and HDI, a new peak at 1571 cm^−1^ was observed in the spectrum of 40%VPU_OH_ due to the formation of −NHCOO groups. Besides, the peak at 3458 cm^−1^ in the spectrum of PEMPA was red-shifted to 3405 cm^−1^ in the spectrum of 40%VPU_OH_ due to the formation of OH⋯NH, OH⋯O=C, or NH⋯O=C hydrogen bonds [36,37,38,39]. Additionally, the VPU_OH_ series with different HDI contents was measured by FTIR, and the spectra are shown in Figure 2b. Taking the absorption at 1727 cm^−1^ as a reference, the ratio between the intensities of the peaks at 1571 and 1727 cm^−1^ (I(1571/1727) = 0.81) in cured 80%VPU_OH_ increased relative to that for PEMPA (I(1571/1727) = 0) due to the formation of the –NHCOO group. With the HDI content increasing (30–80%), the value (I(1571/1727) increased from 0.41 to 0.81, indicating a larger formation of –NHCOO in 80%VPU_OH_. Besides, the peak at 3405 cm^−1^ was distinctly present in 80%VPU_OH_ spectrum, indicating the existence of an unreacted −OH group in the networks, while the peak was obviously smaller in comparison with that of PEMPA. These observations indicated the occurrence of a chemical reaction between −N=C=O and −OH groups. Moreover, the covalently cross-linked molecular architecture of the prepared VPU_OH_ sample was further confirmed by the fact that it was insoluble in organic solvents.

### 3.2. Mechanical Properties, Thermal Performance, and Dynamic Properties Analysis

**Figure 3:**
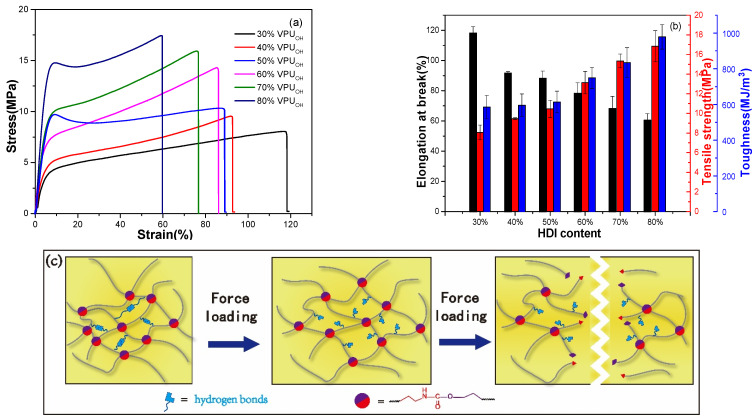
Mechanical properties of PEMPA and the VPU_OH_ series with different HDI content. (**a**) Curve of stress–strain, (**b**) Mechanical properties of the x%VPU_OH_ series with different crosslinker content, (**c**) Schematic representation of the hydrogen bonds.

To investigate the mechanical properties of the VPU_OH_ series, static tensile measurements at a strain rate of 5 s^−1^ were performed. Representative stress–strain curves are displayed in Figure 3a, and the mechanical properties are summarized in Figure 3b and Table 1. It is generally considered that the tensile modulus is closely related to the chain segment’s rigidity and cross-link density of networks [40]. Therefore, the increased cross-linker HDI content could improve the cross-link density, contributing to the enhancement of the tensile strength. As the HDI content increased from 30 to 80%, the tensile strength increased from 8.1 to 16.8 MPa, the toughness increased from 581 to 972 MJ/m^3^, whereas the elongation at break decreased from 119 to 61%, as shown in Table 1. The improved mechanical properties can be attributed to the increased cross-link density and “hard” segments. Compared with the reported PU vitrimer [11,17,20,41], the better mechanical properties of the VPU_OH_ series resulted from the rosin framework with a rigid hydrophenanthrene ring and from the synergistic effect of covalent cross-links and physical cross-links formed by substantial hydrogen bonds. Thus, when stretched, hydrogen bonds fracture firstly dissipated energy, while the covalent bonds maintained a good strength in the VPU_OH_ series, as illustrated in Figure 3c [42]. The covalent bonds control the network flexibility and maintain sample integrity during deformation. On the other hand, the recurrent dissociation/reassociation of the hydrogen bonds in the network controls rigidity and toughness.

**Figure 4:**
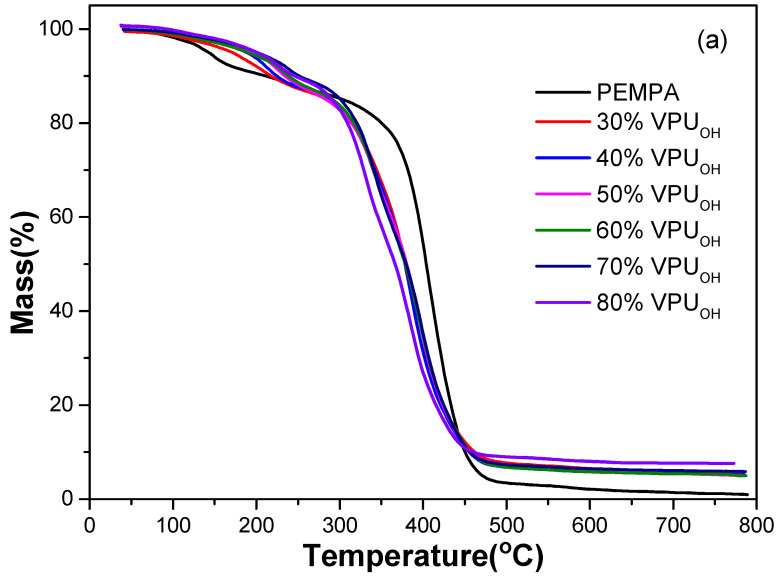

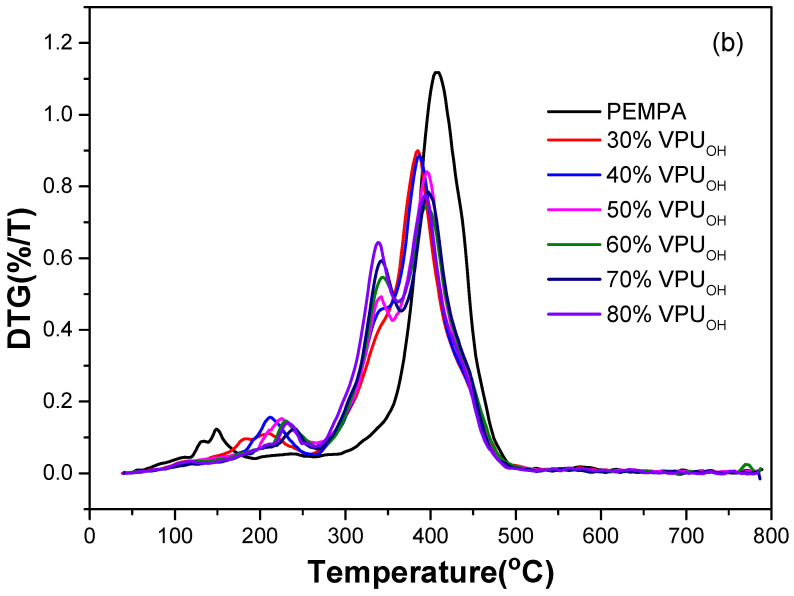
(**a**) TGA and (**b**) DTG curves of PEMPA and the VPU_OH_ series with different HDI content.

TGA at a heating rate of 10 °C/min under a nitrogen atmosphere was performed to study the thermostability of the PEMPA and the VPU_OH_ series with different HDI content. The TGA and DTG curves are shown in Figure 4. PEMPA without HDI showed two obvious weight loss stages around 150 and 430 °C. For the VPU_OH_ series with different content of the HDI crosslinker, the thermal gravimetric profiles were similar, with three obvious weight loss stages around 210, 330, and 400 °C. New weight loss stages around 330 °C probably originated from the –NHCOO bond cleavage in the VPU_OH_ series. Thermal stability factors, including initial decomposition temperature (the temperature of 5 wt% weight loss, T_5d_) and the temperature of 10 wt% weight loss (T_10d_), were determined by TGA. It was observed that the T_5d_ and T_10d_ values of the VPU_OH_ series increased as the HDI content increased, and the initial decomposition temperatures T_5d_ and T_10d_ of the VPU_OH_ series (HDI content 30–80%) increased from 176 to 199 °C and 222 to 249 °C, respectively, because the formation of a network could protect the ester bond adjacent to the rosin framework, contributing to the increased T_5d_ and T_10d_ values. Thermal stability of the VPU_OH_ series prominently improved, and the superior thermal stability of the VPU_OH_ was attributed to the enhanced covalent crosslinker at high HDI content and rigid rosin framework [43]. In all conditions, the VPU_OH_ series exhibited excellent thermal stability, with an onset decomposition temperature of around 200 °C, which is higher than the temperature of thermal processing (~160 °C), demonstrating thermal stability during processing.

**Figure 5:**
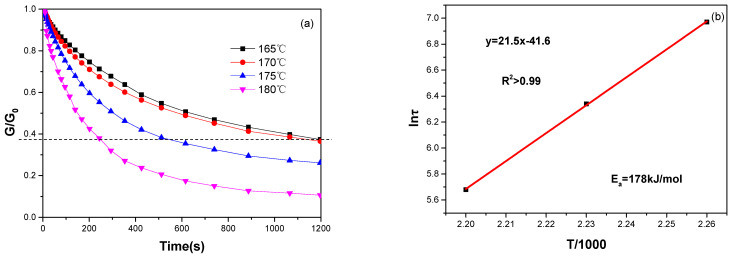

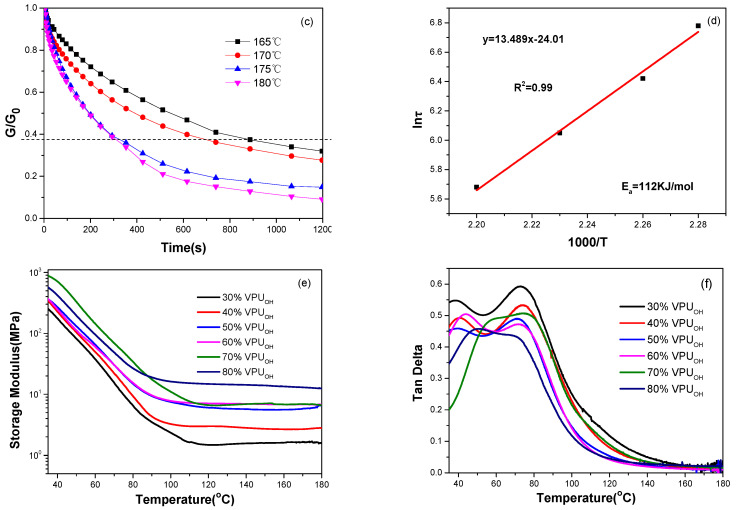
(**a**) Stress relaxation study of 40%VPU_OH_, (**b**) Arrhenius plot with linear fit for 40%VPU_OH_, (**c**) Stress relaxation study of 70%VPU_OH_, (**d**) Arrhenius plot with linear fit for 70%VPU_OH_, (**e**) Storage module curves, and (**f**) Tan δ curves of the VPU_OH_ series with different HDI content.

Thermal stress relaxation tests were employed to study the dynamic structural characteristics of VPU_OH_. The normalized relaxation modulus G/G_0_ of 40%VPU_OH_ and 70%VPU_OH_ is shown as a function of time at different temperatures in Figure 5a,c. When the temperature was elevated to more than 180 and 170 °C, 40%VPU_OH_ and 70%VPU_OH_ quickly relaxed. The curves of stress relaxation of 70%VPU_OH_ at 175 and 180 °C were observed to cross; this might be attributed to a slight fluctuation of the DMA measurement, and similar phenomena were reported previously [44,45,46]. Moreover, the curves of stress relaxation of 70%VPU_OH_ were comparable in the initial stage at 175 and 180 °C. According to Maxwell’s viscoelastic fluid model, the relaxation time (τ) is defined as the time when the sample is relaxed to 1/e of the initial modulus [47]. As shown in Figure 5a,c, the stress relaxation of both 40%VPU_OH_ and 70%VPU_OH_ at 165 °C was slow, and their τ value at 165 °C was estimated to be more than 1200 and around 900 s, respectively, by extrapolating the curve of the relaxation modulus versus time. Normally, the τ values decrease with the elevation of the temperature, due to the increased exchange rate of transcarbamoylation reactions. The activation energy (*E_a_*) of transcarbamoylation exchange reactions can be calculated by the Arrhenius’ law [44,48], following Equation (1), where τ is the relaxation time, *τ*_0_ is the characteristic relaxation time at infinite temperature, T is the testing temperature, and R is the universal gas constant. The Arrhenius relationship of ln (τ) versus 1000/T is shown in Figure 5b,d. The calculated *E_a_* was 178 and 112 kJ mol^−1^ for the 40%VPU_OH_ and 70%VPU_OH_, respectively, which is comparable to that (110, 111, or 184 kJ mol^−1^) of the PU vitrimer with an associative mechanism [14,18,19]. This high activation energy indicated a large difference in the relaxation time at the given temperatures, which is beneficial, for it allowed us to activate and suppress the dynamic exchange in a narrow temperature range. Besides, *E_a_* of 70%VPU_OH_ was obviously lower than that of 40%VPU_OH_ due to the presence of more urethane linkages.

(1) $$\ln\tau =\ln\tau_{0}+\frac{E_{a}}{RT}$$

The topology freezing transition temperature (*T_v_*) is an important characteristic parameter for VPU_OH_. *T_v_* is a hypothetical temperature at which vitrimers convert from solid to liquid and acquire a viscosity of 10^12^ Pa·s [49]. It is generally accepted that the crosslinking networks of vitrimers would freeze when the temperature is below *T_v_*, owing to the low exchange reaction rate. It was determined to be 115 and 91 °C for 40%VPU_OH_ and 70%VPU_OH_, by extrapolation from the Arrhenius’ fitted line, as shown in Figure 5b,d, to a relaxation time of 1 × 10^6^ and 4.1 × 10^5^ s, respectively. When the temperature was above their *T_v_* due to the rapid occurrence of the exchange reactions, both 40%VPU_OH_ and 70%VPU_OH_ networks showed “fluidity”, and the strain sharply increased. In contrast, below their *T_v_*, both 40%VPU_OH_ and 70%VPU_OH_ exhibited a similar performance as ordinary thermosets. Therefore, *T_v_* is also the solidification transition temperature of a topological network. Besides, the VPU_OH_ series displayed only slight stress relaxation around *T_v_*, since its network was frozen by the lack of segmental motions associated with a higher *T_g_*. Therefore, when the temperature was higher than *T_v_* and *T_g_*, networks rearrangement of VPU_OH_ series occurred. Additionally, we observed that with the increase of the HDI content, *E_a_* and *T_v_* decreased due to the presence of more dynamic urethane linkages.

The effects of the HDI content on the storage modulus and α-relaxation of the VPU_OH_ series were studied. The plots of storage modulus and tan δ for the VPU_OH_ series with different HDI contents are shown in Figure 5e,f, and the data are summarized in Table 1. Obviously, VPU_OH_ with a higher HDI content displayed a higher storage modulus at the same temperature, and 80%VPU_OH_ showed the highest rubber platform (*E*′ = 15.3 MPa), as shown in Table 1. This phenomenon should be related to the crosslink density of the VPU_OH_ series; the VPU_OH_ with higher HDI content provided a better-developed network structure which restricted chain mobility and thus enhanced the elastic response of the network. The rubber elasticity equation was used to calculate the crosslink density (*V_e_*) of the VPU_OH_ series (Equation (2)):

*E*′ = 3*RTV_e_* (2)

where the rubbery platform modulus (*E*′) is the storage modulus at the temperature of T (30 °C above *T_g_*), R is the universal gas constant, and *V_e_* is the crosslink density [50]. The cross-link density of cross-linked networks closely relate to their rubbery platform modulus. As shown in Table 1, with the HDI content (30–80%) increasing, the rubbery platform modulus increased from 1.6 to 15.3 MPa, and the Ve increased from 1.7 × 10^−4^ to 16.1 × 10^−4^ mol·m^−3^. Therefore, the enhanced storage modulus originated from the increasing HDI content [51]. Additionally, the typical tan δ plot of VPU_OH_ showed the loss of two peaks at round 43 °C and 78 °C, as shown in Figure 5f, which was possibly due to the α-relaxation of soft and hard segments in the VPU_OH_ network, respectively, because of a phase separation [52,53]. The glass transition temperature (*T_g_*) is another significant parameter for network movements, below which the chain of a network is frozen. Herein it was determined by the α-relaxation of hard segments in VPU_OH_. It is interesting that *T_g_* moved to a lower temperature range when the HDI content of the VPU_OH_ series increased. We observed that the *T_g_* of the VPU_OH_ series increased from 69.3 to 72.7 °C with the HDI contents increasing, because the introduction of the flexible HDI contributed to decreasing the rigidity of the VPU_OH_ networks, as shown in Table 1.

### 3.3. Self-Healing, Welding, and Shape Memory

**Figure 6:**
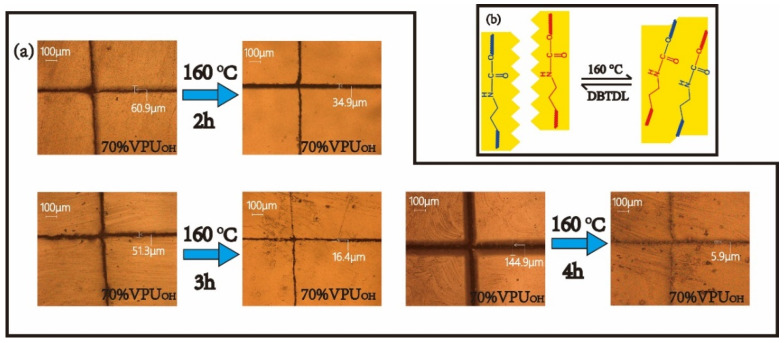
(**a**) Optical microscope images of the self-healing performance of 70%VPU_OH_, (**b**) Schematic representation of the transcarbamoylation exchange reaction.

Network rearrangement and bond reshuffling can take place, thus covalent bonding can be re-established across the interfaces of fractured surfaces, because of the transcarbamoylation exchange reaction of urethane linkages. As a result, 70%VPU_OH_ as a typical example, should acquire self-healing ability by transcarbamoylation exchange-induced network rearrangement. The 70%VPU_OH_ strip sample with a thickness of 0.7 mm was cut using a razor to examine its self-healing capability, as shown in Figure 6a. The cut sample was subjected to a healing treatment at 160 °C for different times (2, 3, or 4 h) in an oven. As shown in Figure 6b, 70%VPU_OH_ exhibited good self-healing capabilities after the healing treatment for 4 h due to the dynamic urethane linkages in the network.

**Figure 7:**
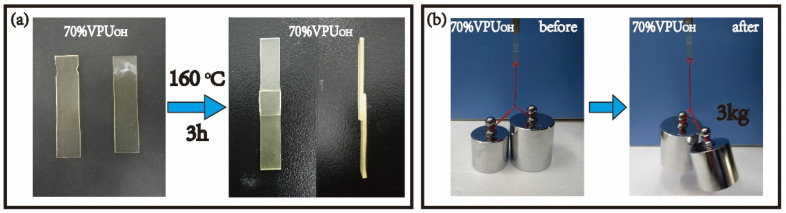
(**a**) Optical images, before and after welding, of 70%VPU_OH_, (**b**) Optical images of the welding performance of 70%VPU_OH_.

To investigate the weldability of 70%VPU_OH_, two rectangular samples (25.0 mm × 5.0 mm × 1.0 mm) were superimposed for a length of 8 mm, as shown in Figure 7a, and held together for the welding time of 3 h at 160 °C. An extensive contact was ensured by applying a compression during the treatment. After the welding test, an integral material could bear more than 3 kg weight without breaking at the healed part, as shown in Figure 7b, indicating a good weldability. The present results demonstrate that the VPU_OH_ network with the dynamic urethane linkages possesses an attractive thermal healing and welding capability.

**Figure 8:**
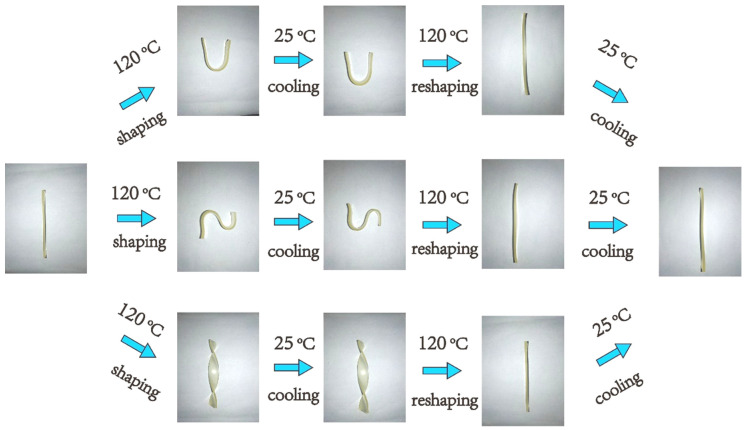
Optical images of the shape-memory performance of 70%VPU_OH_.

The VPU_OH_ vitrimers can be welded and healed at elevated temperature due to transcarbamoylation exchange reactions. Similarly, when the temperature is elevated, VPU_OH_ should show shape memory, because VPU_OH_ can undergo transcarbamoylation exchange reactions to achieve topological rearrangement [48,54]. Figure 8 provides optical images of the shape memory process. At the beginning, 70%VPU_OH_ samples with a permanent “S–U” or oblique shape were deformed above *T_g_* (120 °C). Upon cooling to room temperature, the temporary shapes of the samples were fixed. Subsequently, the temperature was increased to 120 °C again, and the “S–U” or oblique shape reverted to the original flat state within 1 min. It was reported that thermal responsive shape memory property of polymer requires a structure of chemical/physical cross-linking networks and a suitable ratio of stiff and flexible segments [55]. Similarly, the VPU_OH_ network should contain chemical/physical cross-linking and “hard” and “soft” segments, which is in high agreement with the FTIR measurements.

### 3.4. Reprocessing

**Figure 9:**
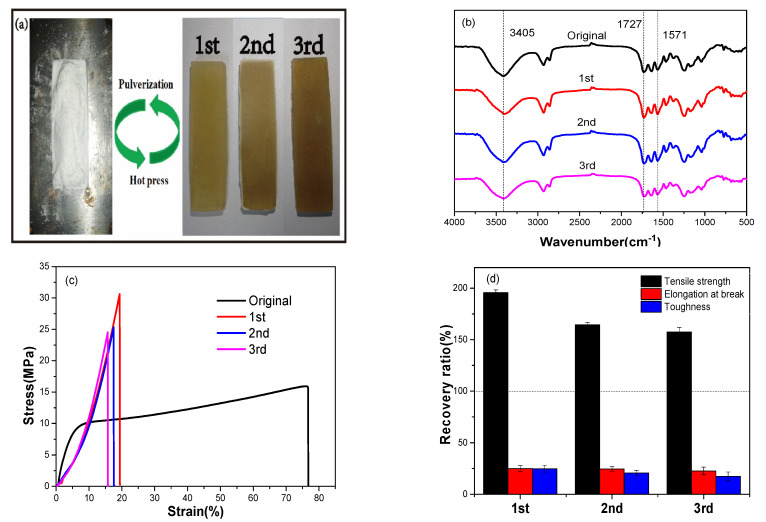

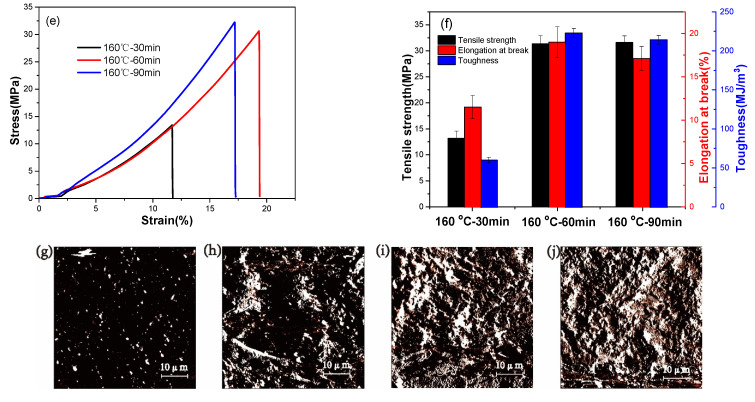
(**a**) Optical images of thermal recycling performance of 70%VPU_OH_ by hot press, (**b**) FTIR spectra of the samples with different cycles of reprocessing, (**c**) Tensile curves and (**d**) Mechanical properties of the samples after different treating time at 160 °C, (**e**) Tensile curves and (**f**) Mechanical properties of the samples for different cycles of reprocessing. AFM phase maps of (**g**) original, (**h**) first-time recycled, (**i**) second-time recycled, and (**j**) third-time recycled 70%VPU_OH_ samples.

To further evaluate the reprocessability of the rosin-based PU vitrimer, 70%VPU_OH_ was pulverized and then was reprocessed by hot pressing to recover the original shape at 160 °C under 5 MPa for 30, 60, or 90 min. Homogeneous samples were obtained after recycling for three times, as shown in Figure 9a. To quantify and qualify the reprocessability, the mechanical properties and FTIR measurement of the original and multiple recycled samples were examined. The characteristic peaks at 3405, 1727, and 1571 cm^−1^ in the FTIR spectra before and after reprocessing several times (Figure 9b) which were assigned to vibrations of −OH, C=O, and −NH, respectively, did not move, indicating chemical structures of the reprocessed and original samples. The cycled samples still maintained an integral and stable crosslinking network. The 70%VPU_OH_ sample showed high thermal stability and remained unchanged, which was due to the nature of the networks with dynamic urethane linkages, consistent with the TGA results. Besides, uniaxial tensile testing was utilized to assess the mechanical properties of multiple reprocessed samples; the stress–strain curves are shown in Figure 9c. The results showed that the pristine sample of 70%VPU_OH_ had an elongation at break of 68.9%, a tensile strength of 15.3 MPa, and a toughness of 828 MJ/m^3^. Typically, the mechanical properties change after the first reprocessing compared with the original properties; tensile strength (32.1 MPa) was distinctly improved, while elongation at break (17.3%) and toughness (216 MJ/m^3^) decreased. Noteworthily, even after the third reprocessing, 70%VPU_OH_ showed a reduction in elongation at break (15.9%) and toughness (144 MJ/m^3^) and an increase in tensile strength (24.2 MPa). The recovery ratios of the mechanical properties for the recycled samples are displayed in Figure 9d. The recovery of tensile strength and elongation at break were more than 196% and 25%, respectively, showing excellent reprocessability, due to the transcarbamoylation exchange reactions [20]. It is noted that tensile strength of all recycled samples was higher than that of the pristine one, maybe because significant phase separation occurred into the soft and hard regions in the pristine sample, after high temperature reprocessing, whereas phase separation was lower in the cycled samples. Atomic force microscopy mapping was employed to examine the phase structure, as shown in Figure 9e,f. The pristine sample showed an island-like phase structure with obvious microphase separation (Figure 9e), and the hard segments appeared dispersed among the soft segments with continuous distribution, which is consistent with the DMA observation that two peaks appeared in the tan δ plot. In contrast, in the three recycled samples, the highlight regions of hard segments increased, indicating improved microphase separation in the recycled samples [56,57,58]. Besides, the effect of the hot press thermal treatment time on the mechanical properties of 70%VPU_OH_ was investigated. The strain–stress curves shown in Figure 9e,f. It was observed that when prolonging the thermal treating time, the mechanical properties were enhanced; however, when the treating time was longer than 60 min, the enhancement of the mechanical properties is not obvious, indicating that the a longer heat treatment time provides improvements only within certain limits, as reported previously [59,60]. In brief, the obtained VPU_OH_ network can serve as a high-performance elastomer or a smart material in the technology field.

## 4. Conclusions

In this work, a rosin-based PU vitrimer with abundant hydroxy groups and dynamic urethane linkages was synthesized. The VPU_OH_ networks showed excellent mechanical (tensile strength of 16.8 MPa and elongation at break of 61%, high toughness 972 MJ/m^3^) and thermal properties (T_5d_ ~200 °C), which were obviously improved in comparison with the monomer PEMPA. The dynamic VPU_OH_ networks possessed *E_a_* around 112–178 kJ·mol^−1^ and *T_v_* around 91–115 °C, and the dynamic performance of the VPU_OH_ network could be regulated by changing the HDI content. Moreover, VPU_OH_ exhibited superior malleability and reprocessability. It was shown that it can be recycled by hot pressing (at 160 °C) due to the transcarbamoylation exchange reactions; and the reprocessed VPU_OH_ maintains network structure and mechanical properties. Even the tensile strength of the recycled samples was higher than the one of the original sample, due to the enhancement of the phase structure in the recycled samples. Because of transcarbamoylation exchange reactions in the VPU_OH_ networks, shape memory and welding and self-heading capabilities were demonstrated also at elevated temperature (120–160 °C).

## Figures and Tables

**Table 1:** Mechanical and thermal properties of the VPU_OH_ series with different HDI content.

Sample	*T_g_* (°C)	*E*′ (*T_g_* + 30 °C) (MPa)	*V_e_* (mol/cm^3^)	Elongation at Break (%)	Tensile Strength (MPa)	Toughness (MJ/m^3^)	Young’s Modulus (MPa)	T_5d_ (°C)	T_10d_ (°C)
30%VPU_OH_	72.7	1.6	1.7 × 10^−4^	119 ± 4.1	8.1 ± 0.8	581 ± 65.1	0.49 ± 0.02	176	222
40%VPU_OH_	75.2	3.0	3.1 × 10^−4^	92 ± 1.0	9.5 ± 0.1	592 ± 62.1	0.64 ± 0.04	187	224
50%VPU_OH_	71.0	6.5	6.8 × 10^−4^	89 ± 4.7	10.5 ± 0.9	609 ± 60.2	0.78 ± 0.04	188	230
60%VPU_OH_	71.2	7.2	7.5 × 10^−4^	84 ± 6.8	13.1 ± 1.2	743 ± 59.5	0.98 ± 0.05	191	237
70%VPU_OH_	72.1	7.3	7.6 × 10^−4^	69 ± 7.9	15.4 ± 0.7	828 ± 82.7	1.19 ± 0.05	198	251
80%VPU_OH_	69.3	15.3	16.1 × 10^−4^	61 ± 4.2	16.8 ± 1.6	972 ± 68.2	2.43 ± 0.06	199	249

## References

[1] Akindoyo J.O., Beg M.D.H., Ghazali S., Islam M.R., Jeyaratnam N., Yuvaraj A.R. (2016). Polyurethane types, synthesis and applications—A review. RSC Adv..

[2] Engels H.W., Pirkl H.G., Albers R., Albach R.W., Krause J., Hoffmann A., Casselmann H., Dormish J. (2013). Polyurethanes: Versatile materials and sustainable problem solvers for today’s challenges. Angew. Chem..

[3] Beran R., Zarybnicka L., Machova D. (2020). Recycling of rigid polyurethane foam: Micro-milled powder used as active filler in polyurethane adhesives. J. Appl. Polym. Sci..

[4] Rivero G., Nguyen L.-T.T., Hillewaere X.K.D., Du Prez F.E. (2014). One-Pot Thermo-Remendable Shape Memory Polyurethanes. Macromolecules.

[5] Lyon G.B., Baranek A., Bowman C.N. (2016). Scaffolded Thermally Remendable Hybrid Polymer Networks. Adv. Funct. Mater..

[6] Heo Y., Sodano H.A. (2014). Self-Healing Polyurethanes with Shape Recovery. Adv. Funct. Mater..

[7] Zhang Y., Ying H., Hart K.R., Wu Y., Hsu A.J., Coppola A.M., Kim T.A., Yang K., Sottos N.R., White S.R. (2016). Malleable and Recyclable Poly(urea-urethane) Thermosets bearing Hindered Urea Bonds. Adv. Mater..

[8] Zhou Z., Zeng Y., Yu C., Chen H., Zhang F. (2020). Mechanically robust, intrinsically self-healing crosslinked polymer enabled by dynamic urea bond exchange reaction. Smart Mater. Struct..

[9] Zhang Z.P., Rong M.Z., Zhang M.Q. (2018). Mechanically Robust, Self-Healable, and Highly Stretchable “Living” Crosslinked Polyurethane Based on a Reversible C–C Bond. Adv. Funct. Mater..

[10] Wang X.-Z., Lu M.-S., Zeng J.-B., Weng Y., Li Y.-D. (2021). Malleable and thermally recyclable polyurethane foam. Green Chem..

[11] Ying W.B., Yu Z., Kim D.H., Lee K.J., Hu H., Liu Y., Kong Z., Wang K., Shang J., Zhang R. (2020). Waterproof, Highly Tough, and Fast Self-Healing Polyurethane for Durable Electronic Skin. ACS Appl. Mater. Interfaces.

[12] Fan W., Jin Y., Shi L., Zhou R., Du W. (2020). Developing visible-light-induced dynamic aromatic Schiff base bonds for room-temperature self-healable and reprocessable waterborne polyurethanes with high mechanical properties. J. Mater. Chem..

[13] Feng Z., Yu B., Hu J., Zuo H., Li J., Sun H., Ning N., Tian M., Zhang L. (2019). Multifunctional Vitrimer-Like Polydimethylsiloxane (PDMS): Recyclable, Self-Healable, and Water-Driven Malleable Covalent Networks Based on Dynamic Imine Bond. Ind. Eng. Chem. Res..

[14] Fortman D.J., Brutman J.P., Cramer C.J., Hillmyer M.A., Dichtel W.R. (2015). Mechanically activated, catalyst-free polyhydroxyurethane vitrimers. J. Am. Chem. Soc..

[15] Zheng N., Fang Z., Zou W., Zhao Q., Xie T. (2016). Thermoset Shape-Memory Polyurethane with Intrinsic Plasticity Enabled by Transcarbamoylation. Angew. Chem..

[16] Kuhl N., Abend M., Geitner R., Vitz J., Zechel S., Schmitt M., Popp J., Schubert U.S., Hager M.D. (2018). Urethanes as reversible covalent moieties in self-healing polymers. Eur. Polym. J..

[17] Bonab V.S., Karimkhani V., Manas-Zloczower I. (2018). Ultra-Fast Microwave Assisted Self-Healing of Covalent Adaptive Polyurethane Networks with Carbon Nanotubes. Macromol. Mater. Eng..

[18] Zheng N., Hou J., Xu Y., Fang Z., Zou W., Zhao Q., Xie T. (2017). Catalyst-Free Thermoset Polyurethane with Permanent Shape Reconfigurability and Highly Tunable Triple-Shape Memory Performance. ACS Macro Lett..

[19] Wen Z., McBride M.K., Zhang X., Han X., Martinez A.M., Shao R., Zhu C., Visvanathan R., Clark N.A., Wang Y. (2018). Reconfigurable LC Elastomers: Using a Thermally Programmable Monodomain To Access Two-Way Free-Standing Multiple Shape Memory Polymers. Macromolecules.

[20] Chen X., Li L., Jin K., Torkelson J.M. (2017). Reprocessable polyhydroxyurethane networks exhibiting full property recovery and concurrent associative and dissociative dynamic chemistry via transcarbamoylation and reversible cyclic carbonate aminolysis. Polym. Chem..

[21] Fortman D., Brutman J.P., Hillmyer M.A., Dichtel W.R. (2017). Structural effects on the reprocessability and stress relaxation of crosslinked polyhydroxyurethanes. J. Appl. Polym. Sci..

[22] Bossion A., Heifferon K.V., Meabe L., Zivic N., Taton D., Hedrick J.L., Long T.E., Sardon H. (2019). Opportunities for organocatalysis in polymer synthesis via step-growth methods. Prog. Polym. Sci..

[23] Fortman D.J., Sheppard D.T., Dichtel W.R. (2019). Reprocessing Cross-Linked Polyurethanes by Catalyzing Carbamate Exchange. Macromolecules.

[24] Yan P., Zhao W., Fu X., Liu Z., Kong W., Zhou C., Lei J. (2017). Multifunctional polyurethane-vitrimers completely based on transcarbamoylation of carbamates: Thermally-induced dual-shape memory effect and self-welding. RSC Adv..

[25] Liu W., Fang C., Wang S., Huang J., Qiu X. (2019). High-Performance Lignin-Containing Polyurethane Elastomers with Dynamic Covalent Polymer Networks. Macromolecules.

[26] Xu X., Song Z., Shang S., Cui S., Rao X. (2011). Synthesis and properties of novel rosin-based water-borne polyurethane. Polym. Int..

[27] Ma Q., Liu X., Zhang R., Zhu J., Jiang Y. (2013). Synthesis and properties of full bio-based thermosetting resins from rosin acid and soybean oil: The role of rosin acid derivatives. Green Chem..

[28] Yang X., Guo L., Xu X., Shang S., Liu H. (2020). A fully bio-based epoxy vitrimer: Self-healing, triple-shape memory and reprocessing triggered by dynamic covalent bond exchange. Mater. Des..

[29] Liu X., Xin W., Zhang J. (2009). Rosin-based acid anhydrides as alternatives to petrochemical curing agents. Green Chem..

[30] Wang H., Liu X., Liu B., Zhang J., Xian M. (2009). Synthesis of rosin-based flexible anhydride-type curing agents and properties of the cured epoxy. Polym. Int..

[31] Li Z., Yang X., Liu H., Yang X., Shan Y., Xu X., Shang S., Song Z. (2019). Dual-functional antimicrobial coating based on a quaternary ammonium salt from rosin acid with in vitro and in vivo antimicrobial and antifouling properties. Chem. Eng. J..

[32] Thakur T., Jaswal S., Parihar S., Gaur B., Singha A.S. (2020). Bio-based epoxy thermosets with rosin derived imidoamine curing agents and their structure-property relationships. Express Polym. Lett..

[33] Deng L., Ha C., Sun C., Zhou B., Yu J., Shen M., Mo J. (2013). Properties of Bio-based Epoxy Resins from Rosin with Different Flexible Chains. Ind. Eng. Chem. Res..

[34] Hu J., Chen Z., He Y., Huang H., Zhang X. (2016). Synthesis and structure investigation of hexamethylene diisocyanate (HDI)-based polyisocyanates. Res. Chem. Intermed..

[35] Cornejo J.J.M., Matsuoka E., Daiguji H. (2011). Size control of hollow poly-allylamine hydrochloride/poly-sodium styrene sulfonate microcapsules using the bubble template method. Soft Matter.

[36] Liu M., Zhong J., Li Z., Rong J., Yang K., Zhou J., Shen L., Gao F., Huang X., He H. (2020). A high stiffness and self-healable polyurethane based on disulfide bonds and hydrogen bonding. Eur. Polym. J..

[37] Tien Y.I., Wei K.H. (2001). Hydrogen bonding and mechanical properties in segmented montmorillonite/polyurethane nanocomposites of different hard segment ratios. Polymer.

[38] Wang S., Teng N., Dai J., Liu J., Cao L., Zhao W., Liu X. (2020). Taking advantages of intramolecular hydrogen bonding to prepare mechanically robust and catalyst-free vitrimer. Polymer.

[39] Wang C.F., Su Y.C., Kuo S.W., Huang C.F., Sheen Y.C., Chang F.C. (2006). Low-surface-free-energy materials based on polybenzoxazines. Angew. Chem..

[40] Wang S., Ma S., Li Q., Yuan W., Wang B., Zhu J. (2018). Robust, Fire-Safe, Monomer-Recovery, Highly Malleable Thermosets from Renewable Bioresources. Macromolecules.

[41] Yan P., Zhao W., Wang Y., Jiang Y., Zhou C., Lei J. (2017). Carbon Nanotubes-Polyurethane Vitrimer Nanocomposites with the Ability of Surface Welding Controlled by Heat and Near-Infrared Light. Macromol. Chem. Phys..

[42] Liu Y., Tang Z., Wu S., Guo B. (2019). Integrating Sacrificial Bonds into Dynamic Covalent Networks toward Mechanically Robust and Malleable Elastomers. ACS Macro Lett..

[43] Yang X., Li Q., Li Z., Xu X., Liu H., Shang S., Song Z. (2019). Preparation and Characterization of Room-Temperature-Vulcanized Silicone Rubber Using Acrylpimaric Acid-Modified Aminopropyltriethoxysilane as a Cross-Linking Agent. ACS Sustain. Chem. Eng..

[44] Ruiz de Luzuriaga A., Martin R., Markaide N., Rekondo A., Cabañero G., Rodríguez J., Odriozola I. (2016). Epoxy resin with exchangeable disulfide crosslinks to obtain reprocessable, repairable and recyclable fiber-reinforced thermoset composites. Mater. Horiz..

[45] Zhou F., Guo Z., Wang W., Lei X., Zhang B., Zhang H., Zhang Q. (2018). Preparation of self-healing, recyclable epoxy resins and low-electrical resistance composites based on double-disulfide bond exchange. Compos. Sci. Technol..

[46] Zhang L., Rowan S.J. (2017). Effect of Sterics and Degree of Cross-Linking on the Mechanical Properties of Dynamic Poly(alkylurea–urethane) Networks. Macromolecules.

[47] Montarnal D., Capelot M., Tournilhac F., Leibler L. (2011). Silica-Like Malleable Materials from Permanent Organic Networks. Science.

[48] Liu T., Hao C., Wang L., Li Y., Liu W., Xin J., Zhang J. (2017). Eugenol-Derived Biobased Epoxy: Shape Memory, Repairing, and Recyclability. Macromolecules.

[49] Brutman J.P., Delgado P.A., Hillmyer M.A. (2014). Polylactide Vitrimers. ACS Macro Lett..

[50] Chen J.-H., Yuan W.-Q., Li Y.-D., Weng Y.-X., Zeng J.-B. (2019). Malleable and Sustainable Poly(ester amide) Networks Synthesized via Melt Condensation Polymerization. ACS Sustain. Chem. Eng..

[51] Liu Y.-Y., He J., Li Y.-D., Zhao X.-L., Zeng J.-B. (2020). Biobased, reprocessable and weldable epoxy vitrimers from epoxidized soybean oil. Ind. Crop Prod..

[52] Karimi M.B., Khanbabaei G., Sadeghi G.M.M. (2019). Unsaturated canola oil-based polyol as effective nucleating agent for polyurethane hard segments. J. Polym. Res..

[53] Lligadas G., Ronda J.C., Galià M., Cádiz V. (2007). Poly(ether urethane) networks from renewable resources as candidate biomaterials: Synthesis and characterization. Biomacromolecules.

[54] Hornat C.C., Yang Y., Urban M.W. (2017). Quantitative Predictions of Shape-Memory Effects in Polymers. Adv. Mater..

[55] Xie F., Huang L., Leng J., Liu Y. (2016). Thermoset shape memory polymers and their composites. J. Intell. Mater. Syst. Struct..

[56] Fan X., Zhiping L.V., Xiang Z.U. (2019). Study on microphase separation of novel crosslinked polyurethane by AFM and DMA. Rev. Roum. Chim..

[57] Ling F., Liu Z., Chen M., Wang H., Zhu Y., Ma C., Wu J., Huang G. (2019). Compatibility driven self-strengthening during the radical-responsive remolding process of poly-isoprene vitrimers. J. Mater. Chem..

[58] Zeng Y., Liu S., Xu X., Chen Y., Zhang F. (2020). Fabrication and curing properties of o-cresol formaldehyde epoxy resin with reversible cross-links by dynamic boronic ester bonds. Polymer.

[59] Li H., Zhang B., Yu K., Yuan C., Zhou C., Dunn M.L., Qi H.J., Shi Q., Wei Q.H., Liu J. (2020). Influence of treating parameters on thermomechanical properties of recycled epoxy-acid vitrimers. Soft Matter.

[60] Zhang B., Li H., Yuan C., Dunn M.L., Qi H.J., Yu K., Shi Q., Ge Q. (2020). Influences of processing conditions on mechanical properties of recycled epoxy-anhydride vitrimers. J. Appl. Polym. Sci..

